# TIE-GANs: single-shot quantitative phase imaging using transport of intensity equation with integration of GANs

**DOI:** 10.1117/1.JBO.29.1.016010

**Published:** 2024-01-30

**Authors:** Vikas Thapa, Ashwini Subhash Galande, Gurram Hanu Phani Ram, Renu John

**Affiliations:** Indian Institute of Technology Hyderabad, Medical Optics and Sensors Laboratory, Department of Biomedical Engineering, Hyderabad, Telangana, India

**Keywords:** quantitative phase imaging, transport of intensity equation, generative adversarial networks, phase map, deep learning

## Abstract

**Significance:**

Artificial intelligence (AI) has become a prominent technology in computational imaging over the past decade. The expeditious and label-free characteristics of quantitative phase imaging (QPI) render it a promising contender for AI investigation. Though interferometric methodologies exhibit potential efficacy, their implementation involves complex experimental platforms and computationally intensive reconstruction procedures. Hence, non-interferometric methods, such as transport of intensity equation (TIE), are preferred over interferometric methods.

**Aim:**

TIE method, despite its effectiveness, is tedious as it requires the acquisition of many images at varying defocus planes. The proposed methodology holds the ability to generate a phase image utilizing a single intensity image using generative adversarial networks (GANs). We present a method called TIE-GANs to overcome the multi-shot scheme of conventional TIE.

**Approach:**

The present investigation employs the TIE as a QPI methodology, which necessitates reduced experimental and computational efforts. TIE is being used for the dataset preparation as well. The proposed method captures images from different defocus planes for training. Our approach uses an image-to-image translation technique to produce phase maps and is based on GANs. The main contribution of this work is the introduction of GANs with TIE (TIE:GANs) that can give better phase reconstruction results with shorter computation times. This is the first time the GANs is proposed for TIE phase retrieval.

**Results:**

The characterization of the system was carried out with microbeads of 4  μm size and structural similarity index (SSIM) for microbeads was found to be 0.98. We demonstrated the application of the proposed method with oral cells, which yielded a maximum SSIM value of 0.95. The key characteristics include mean squared error and peak-signal-to-noise ratio values of 140 and 26.42 dB for oral cells and 100 and 28.10 dB for microbeads.

**Conclusions:**

The proposed methodology holds the ability to generate a phase image utilizing a single intensity image. Our method is feasible for digital cytology because of its reported high value of SSIM. Our approach can handle defocused images in such a way that it can take intensity image from any defocus plane within the provided range and able to generate phase map.

## 1 Introduction

Cellular analysis of biological cells is one of the most popular tools for early diagnosis of human diseases. Cytological investigations unveil the latent topographical characteristics of cells, thereby aiding clinicians in making crucial diagnostic decisions concerning diseases.^1^ Cytology became more informative and useful as biology and microscopic methods advanced in conjunction. The invention of the microscope^2^ in 1600 had a profound impact on the scientific community. Conventional brightfield microscopy techniques have the capability to visualize amplitude objects, as they solely exhibit intensity. Transparent objects, such as biological cells and microorganisms, present a challenge for visualization under a brightfield microscope due to their transparent nature.

Staining the cells is considered one of the potential solutions to address this issue. But staining is not a recommended option because it can cause morphological or chemical alterations in the cells. The examination of cells utilizing qualitative feature maps^3^ does not disclose the morphological characteristics of the specimen. Consequently, the utilization of solely intensity-based information does not allow for accurate characterization based on morphology. The label-free high contrast optical imaging of living cells was made possible by Zernike’s invention of the phase contrast microscope (PCM) in 1930.^4^ The Normarski differential interference contrast (DIC) microscope produces phase maps in a similar manner.^5^^,^^6^ Phase contrast techniques, such as PCM and DIC, produce good contrast images but do not reveal thickness information. Quantitative phase imaging (QPI) is a label-free methodology that measures the phase delay caused by the sample,^7^^–^^9^ thereby enhancing the precision of disease diagnosis. The utilization of QPI live cell imaging has allowed researchers to prevent the expenses associated with laborious staining procedures. With subwavelength accuracies, it provides the optical thickness profile of a transparent object, which is vital information for cell biology and meteorology. Although there are several benefits associated with QPI, it continues to be a laborious commitment.

The integration of QPI and deep learning (DL) has demonstrated significant potential in providing diverse applications in recent times. While QPI has the potential to address issues pertaining to precise thickness mapping of biological samples DL is better suited for managing extensive datasets and automating processes.^10^^,^^11^ DL techniques aim to replicate the functionality of biological neurons in the human brain.^12^

Fast phase retrieval using only a single intensity image was initially reported by Rivenson et al.,^13^ demonstrating the power of DL. This method not only solved the problem of reconstruction, but it can generate phase and amplitude images, using only single hologram intensity. However, one needs to be specifically trained to reconstruct the phase and amplitude to eliminate subjective. Uncertainty maps describe shortcomings, such as noise, model error, missing training data, and out-of-sample testing data, that are common in practice but rarely accounted for. DL predictions are not checked for reliability and hence mistakes are often identified only after the results are achieved. Xue et al.^14^ overcome this problem. Lensless imaging is novel in terms of its low cost, portability, and fast retrieval times. A lensless imaging system to recover phase from a single intensity diffraction pattern was proposed by Sinha et al.^15^ as a solution to the inverse computational imaging issue known as “phase retrieval.”^16^^,^^17^ Some of the techniques employ holography with convolutional neural network (CNN) and generative adversarial networks (GANs) to quickly retrieve the phase and amplitude from a single hologram image.^18^^–^^21^ Ptychography^22^^–^^24^ is one of the popular reconstruction techniques for phase recovery. Kaiqiang et al. discussed the TIE with deep learning (termed as dTIE).^25^ The method shows DL ability to produce phase map from single intensity image. The reported average structural similarity index (SSIM) for the proposed method is 0.95. Wu et al. discussed the fundamental principles of phase imaging with the DL-based technique to create a model-based phase retrieval framework.^26^ The low-rank total variation based regularization approach reports a peak-signal-to-noise ratio (PSNR) of 11.63 and an SSIM of 0.92, whereas the model-based network regularization method reports PSNRs of 23.70 and SSIMs of 0.90. Xiaofeng et al. created a cascaded deep neural network using the forward and inverse physics models to create a physics-informed neural network (PINN).^27^ PINN method reports the SSIM value of 0.91 for human buccal epithelial cells 25.23 dB in PSNR value. The extended use of CNN and U-Net to achieve large space bandwidth and high-resolution imaging is possible with ptychography method. The integration of CNN with lensless imaging shows promising results and has clinical applications in real world.^28^^,^^29^ One of the modalities for phase imaging is tomography, which shows high resolution and better performance for voxel reconstruction^30^^,^^31^ using deep convolutional neural network. Transport of intensity equation (TIE) is a non-interferometric QPI technique that enables the reconstruction of phase information from defocused intensity measurements in a regular microscope.^32^ Though the TIE is elegant and simple, the need for multiple defocus measurements and a precise phase characterization prior to use made it unpopular in practical real time use.

In this work, we introduce an efficient method known as TIE-GANs to overcome multi-shot scheme of conventional TIE. The proposed approach is advantageous as it can be implemented in real time as a single shot technique. Our approach is based on the mapping relationship between intensity and phase image. The use of GANs^33^ in the field of computer vision is a groundbreaking work with applications in various operations, such as image-to-image translation,^34^ text-to-image translation,^35^ and semantic image to photo translation.^36^ Image-to-image translation application is exploited in demonstrating virtual phase staining of the images.^37^ This approach makes use of image-to-image translation using GANs modality.^38^ The image-to-image translation method finds the relationship between input and output images. It is unique in terms of loss learning because this approach not just learns image translation from input to output map but also learns a loss function simultaneously. Due to the time and effort required for execution, the network no longer needs to rely on hand-engineered loss functions. For dataset preparation, TIE is being used for creating phase and intensity image pair.^39^ In optical and electron microscopy, the TIE is a computational method for reconstructing the phase of a complex wave.^40^ One of the main bottlenecks for DL algorithms is that it requires a large dataset, i.e., images for proper training. Hence, during the experiments our focus was to generate an appropriate number of images for training the neural network. In this study, we used oral buccal cells for training the GANs-based model. There are two sets of image pairs generated using TIE microscope: the first set consists of 600 and the second one consists of 700 images consecutively. Out of these image stacks, 100 images are reserved for testing from each set while the remaining images are used for training the network. We use SSIM, mean squared error (MSE), PSNR, and universal image quality index (UIQI) as the main image quality assessment parameters to evaluate the translated and reconstructed images.^41^

## 2 Principle

### 2.1 Transport of Intensity Equation

In the recent past, several QPI imaging techniques have been developed for biomedical applications.^42^ In QPI techniques based on interferometric principles, one of the main challenges is to address the coherence induces disturbances^43^ that can hinder the quality and accuracy of phase reconstructions. Traditional intensity-based imaging methods in QPI capture only the object’s intensity information, whereas the phase information, which is related to the object’s refractive index variations, is lost.^44^ The TIE method, which is a non-interferometric QPI technique, enables the phase information to be recovered from intensity measurements.^45^^–^^47^

TIE is based on Poisson equation, which takes into consideration the Laplacian, of the phase of wave and its variation along the optical axis.^48^ The TIE makes use of a well-focused image and two defocused images to extract the complex wave information of the object. A plane wave in terms of amplitude and phase term can be represented as

$$u(x,y)=\sqrt{I(x,y)}\;\exp(i\varphi (x,y)),$$ (1)

where I is the captured intensity and φ(x,y) is the spatial distribution of the phase. Paraxial approximation of the propagation gives the TIE,^49^ as

$$\frac{\partial I(x,y)}{\partial z}=-\frac{\lambda }{2\pi }\nabla .(I\nabla \varphi (x,y)),$$ (2)

where λ represents the spectrally weighted mean wavelength. By slightly defocusing the image in both the positive and negative z-direction z derivative can be obtained. Phase retrieval can be calculated,^50^ using Eq. (2) to compute the inverse Laplacian.

The φ(x,y) can be obtained as

$$\varphi (x,y)=\nabla^{-2}[\nabla \cdot [\frac{1}{I(x,y)}\nabla [\nabla^{-2}(-\frac{2\pi }{\lambda }\frac{\partial I(x,y)}{\partial z})]]],$$ (3)

where I(x,y) represents the infocus image intensity and the $\nabla^{-2}$ is the inverse Laplacian operator.

Finally, to obtain the quantitative measurement of the phase in terms of the thickness of the object h(x,y) can be obtained as

$$h(x,y)=\frac{\lambda }{2\pi \Delta n}\Delta \varphi ,$$ (4)

where Δn represents the refractive index difference.

Our approach utilizes the non-interferometric property of TIE to record multiple image stack for training our model.^51^ TIE methodology is based on multiple recordings at different defocus planes, hence while reconstructing the phase image one must consider one infocus and two defocus images.^52^ Finding infocus image plane could be another difficult task if one must decide manually from a stack of images. We considered Tamura coefficient to calculate the best infocus image so that the reconstructed image has the highest possible SNR.^53^ Training with the wide spectrum of defocus images leads to a robust network and therefore, this modality could reconstruct the object information at larger defocus planes. Our method incorporates about ±60  μm defocus distance hence, a well defocused image can also produce a reliable reconstructed phase image.

### 2.2 Generative Adversarial Networks

DL can extract features from multimodal dataset and recognizes the patterns.^54^ This property makes it suitable for various tasks, such as classification,^55^ cancer detection,^56^ and segmentation.^57^ A DL model is composed of multiple hidden layers, at every layer input is the linear combination of previous layer output, which then passes through a nonlinear function on weighted sums.^58^

Output of k’th node in layer n+1 is denoted as

$$Z_{k}^{\left(n+1\right)}=h(a_{k}^{\left(n+1\right)})=h(\overset{N_{n}}{\underset{i=0}{\sum }}w_{ki}^{\left(n\right)}x_{i}^{\left(n\right)})\;k=0,\ldots ,K;\;n=1,\ldots ,N_{n+1},$$ (5)

which is derived from the input $x_{1}^{n},x_{2}^{n},x_{3}^{n},\ldots .,x_{N}^{n}$.

$a_{k}^{\left(n+1\right)}$ is the linear combination of inputs, and $w_{ki}^{\left(n\right)}$ are weights associated with these inputs these are also known as convolutional maps.^59^ Number of nodes in the layer l to L represents the number of hidden layers; usually a single hidden layer represents a shallow and more than three layers is known as the deep neural network.^60^ The nonlinear activation function choices are sigmoid function $h(a)=\frac{1}{\left(1+\exp(-a)\right)}$, RELU h(a)=max(0,a), and hyperbolic tangent tanh(a) function.^61^

**Fig. 1:**
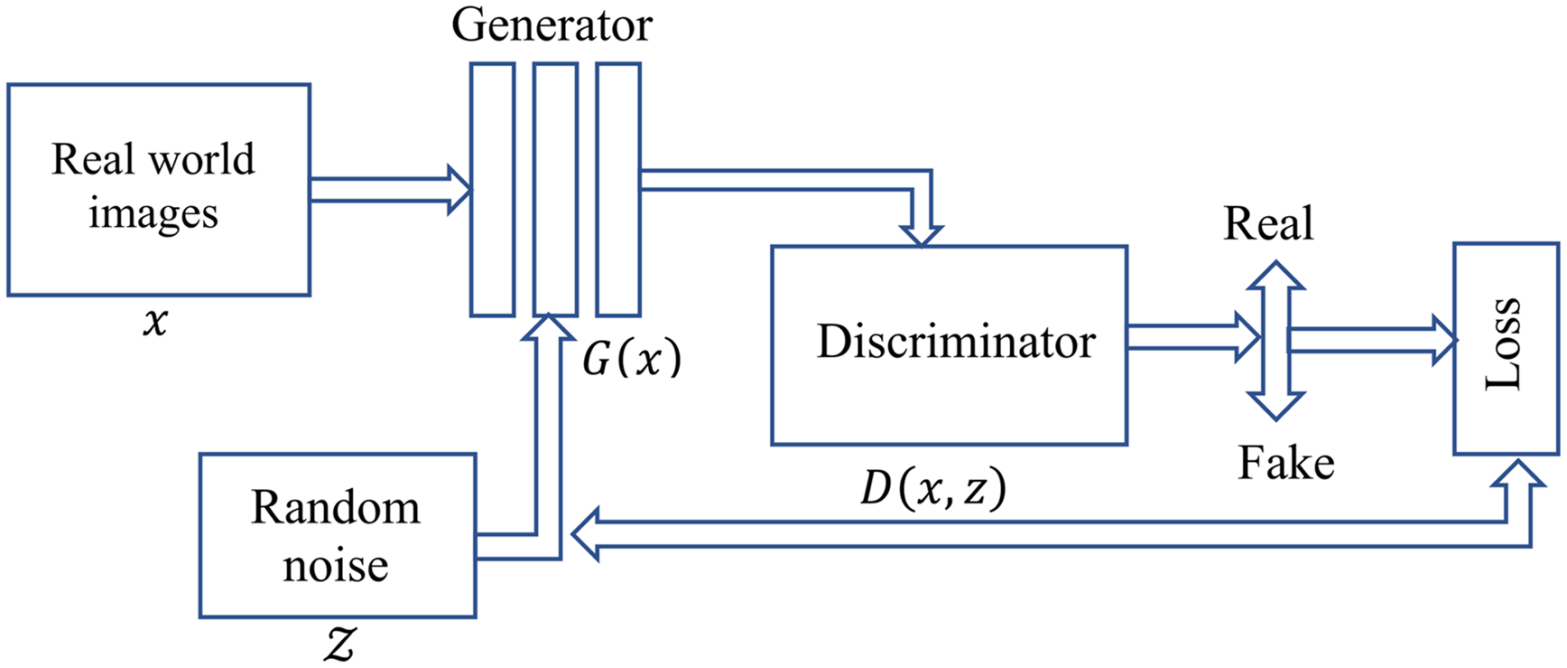
GANs training map.

GANs work on a principle where a model can generate data in an adversarial training setup.^62^ GANs are different from conventional discriminative model where an image is given as X and model try to predict the label Y→P(Y|X). The main disadvantage associated with the discriminative model is that they cannot model P(X), i.e., they are not capable of modeling input images. Hence, these models cannot decide the probability map of input images therefore unable to generate new images. GANs are unique in terms of learning loss, which classify the output images as real or fake and simultaneously train a generative model to minimize this loss. Our approach is focused on a modality know as conditional generative adversarial networks (cGANs). This method is capable of converting an image from one domain to another using conventional GANs in a conditional setting. The main advantage of this technique is that it is best suited for image-to-image translation task in which an input image is conditioned and generates corresponding output image.^63^^,^^64^ The training procedure associated with the GANs is shown in Fig. 1.

Mainly a GAN consists of a generator G and a discriminator D, GAN works as a minmax game theory where discriminator tries to maximize its reward and generator tries to maximize discriminator loss.

GANs learn to convert a random noise vector z to output image map y, G : z→y. In the case of cGANs, it learns mapping from observed images x and random noise vector z, z→y, G:{x,z}→y.

The objective function of conditional GANs is given as

$$L_{\mathrm{cGAN}}(G,D)=E_{x,y}[\log\;D(x,y)]+E_{x,z}[\log(1-D(x,G(x,z)))],$$ (6)

G minimizes the objective function against an adversarial D, which is trying to maximize the cost function.

It can be represented as

$$G^{*}={\arg\;\min}_{G}\;\max_{D}\;L_{\mathrm{cGAN}}(G,D).$$ (7)

The detailed description of this network and method is shown in Figs. 5(a) and 5(b).

## 3 Materials and Methods

### 3.1 Experimental Set-Up

**Fig. 2:**
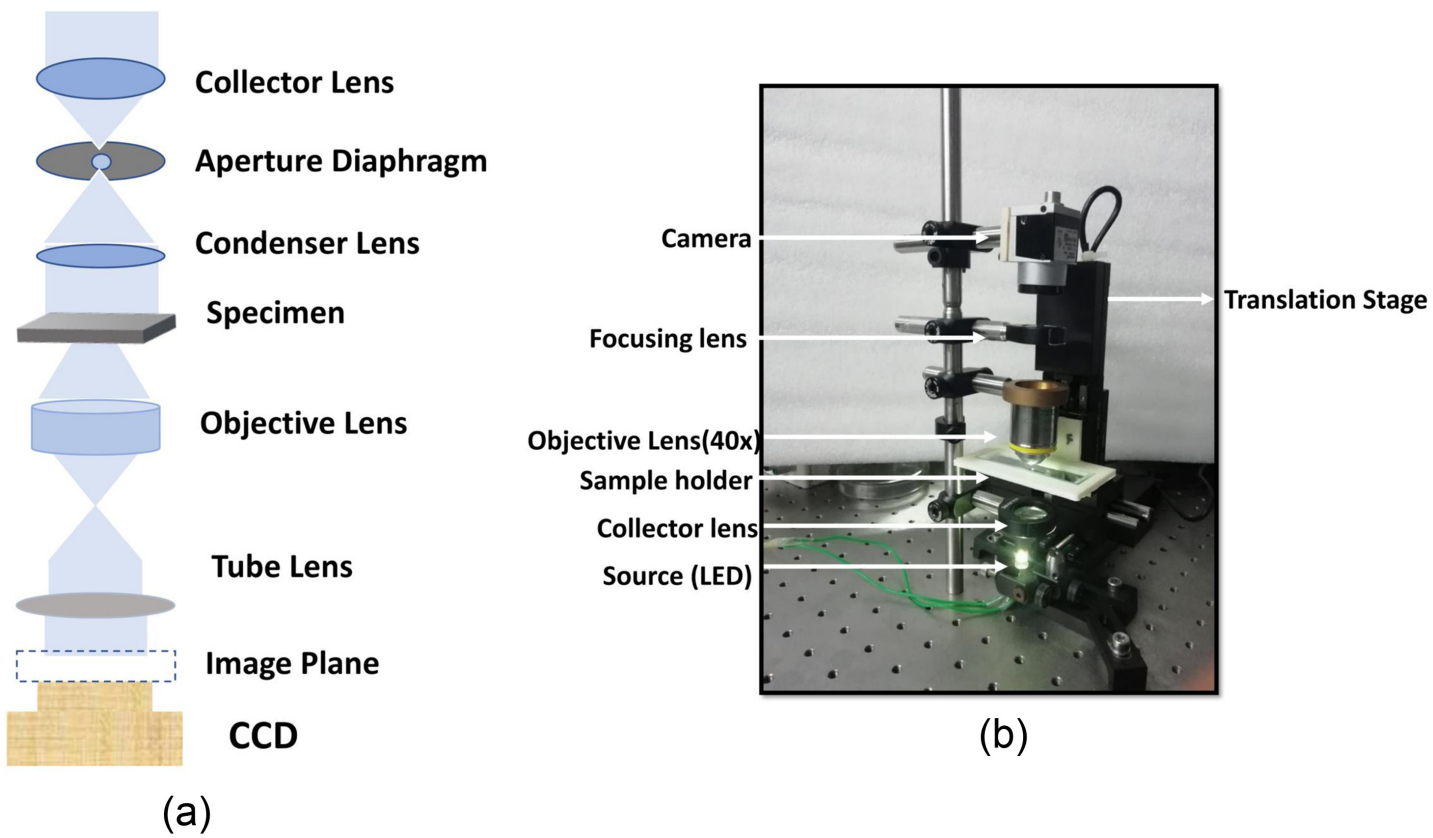
(a) Schematic block diagram of TIE experiment. (b) The optical setup for the experiment.

Oral smears were produced from the buccal mucosa of six healthy 25- to 30-year-old volunteers using a sterile wooden spatula on a clean sterile glass slide. Before taking samples, all of the respondents were asked to gargle with standard saline. After fixing the cell with alcohol, samples were put right on a microscope slide so that images can be recorded. No staining procedures were followed in this process. The oral cells were illuminated using a partially coherent LED light source having wavelength of 627 nm. The camera used for the experiment is (Basler acA2440-75um USB 3.0) with (Sony IMX250 CMOS) sensor of 5 MP resolution operating at a frame rate of 75 fps, with 3.45  μm×3.45  μm pixel size. A microscope objective with 40× magnification and a numerical aperture (NA) of 0.65 is used in the microscope. An automated translation stage (Thorlabs MTS50-Z8) having a travel range of 50 mm with 0.05  μm minimum achievable incremental movement or step size is used for scanning the focus in z direction. The schematic of the experimental set-up for TIE is shown in Fig. 2(a), and the optical setup for the experiment is shown in Fig. 2(b).

The stack of images was recorded with a defocus distance up to ±60  μm and a step size of 15  μm along the Z axis, for creating the training dataset images. This image stack data are further used to train GANs. The captured intensity image is of size 1388×1040  pixels from which a region of interest (ROI) containing each individual cell size of 256×256  pixels was extracted out. Reconstructions were performed on a computer with an Intel Core i9 9820X (10Cores 20Threads up to 4.2 GHz) CPU, 64 GB of RAM, and an NVIDIA RTX 2080Ti Dual GPU setup. The training took between 1 and 1.5 h, based on how many sets of images were used.

### 3.2 Reconstruction Algorithm of TIE

**Fig. 3:**
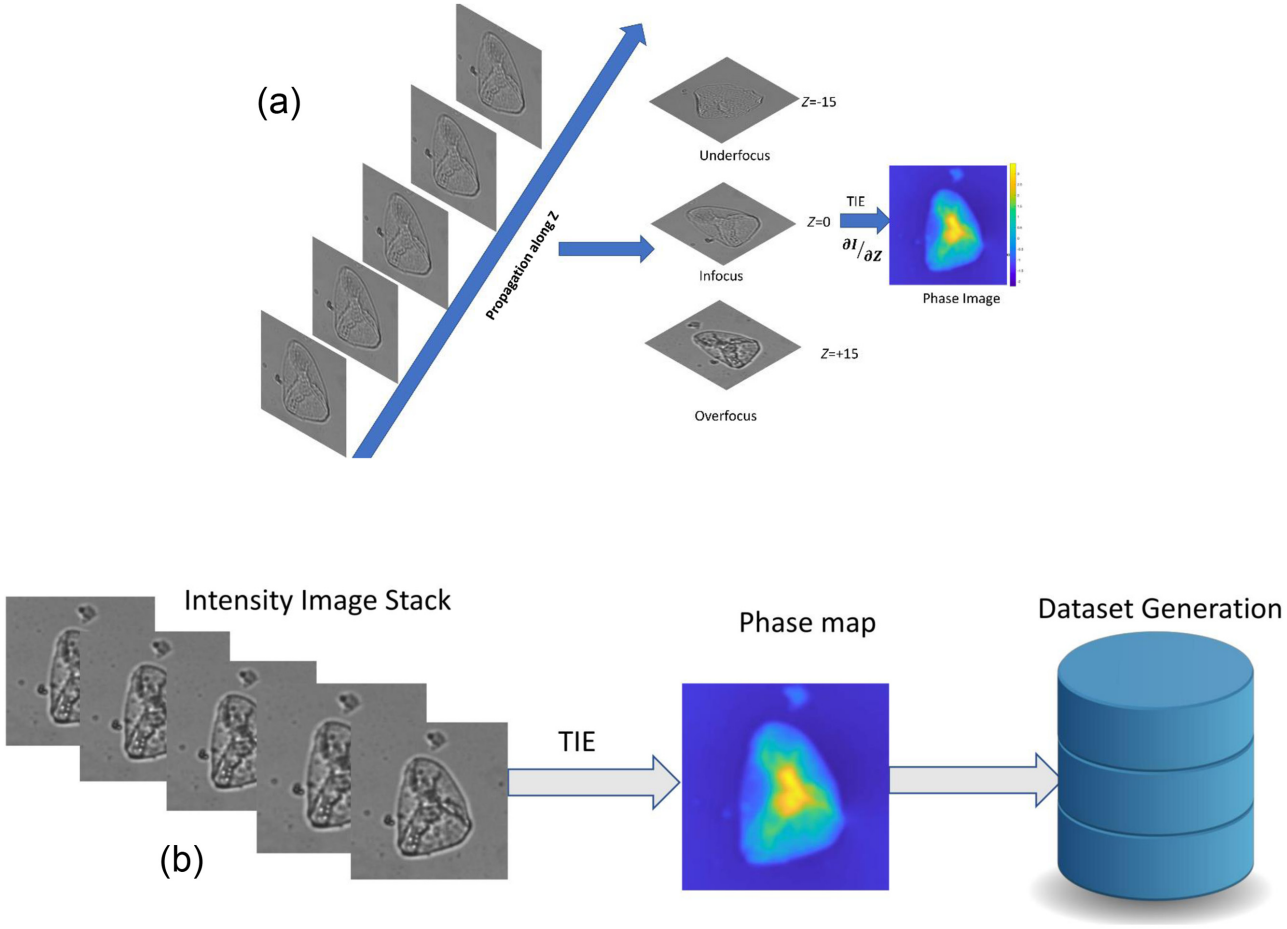
(a) Block diagram of phase extraction using TIE. (b) Dataset generation of phase map using TIE data.

First the imaging stack was created using the TIE microscope as TIE establishes the connection between intensity variation and the phase gradient.^65^ We use the TIE for extraction of the phase from the intensity variation.^66^ Figure 3(a) shows the TIE phase retrieval block diagram.

After phase extraction, the dataset is created using pairs of intensity and phase images, which is given for the training. The methodology diagram for the dataset generation is presented in Fig. 3(b).

### 3.3 TIE-GANs

**Fig. 4:**
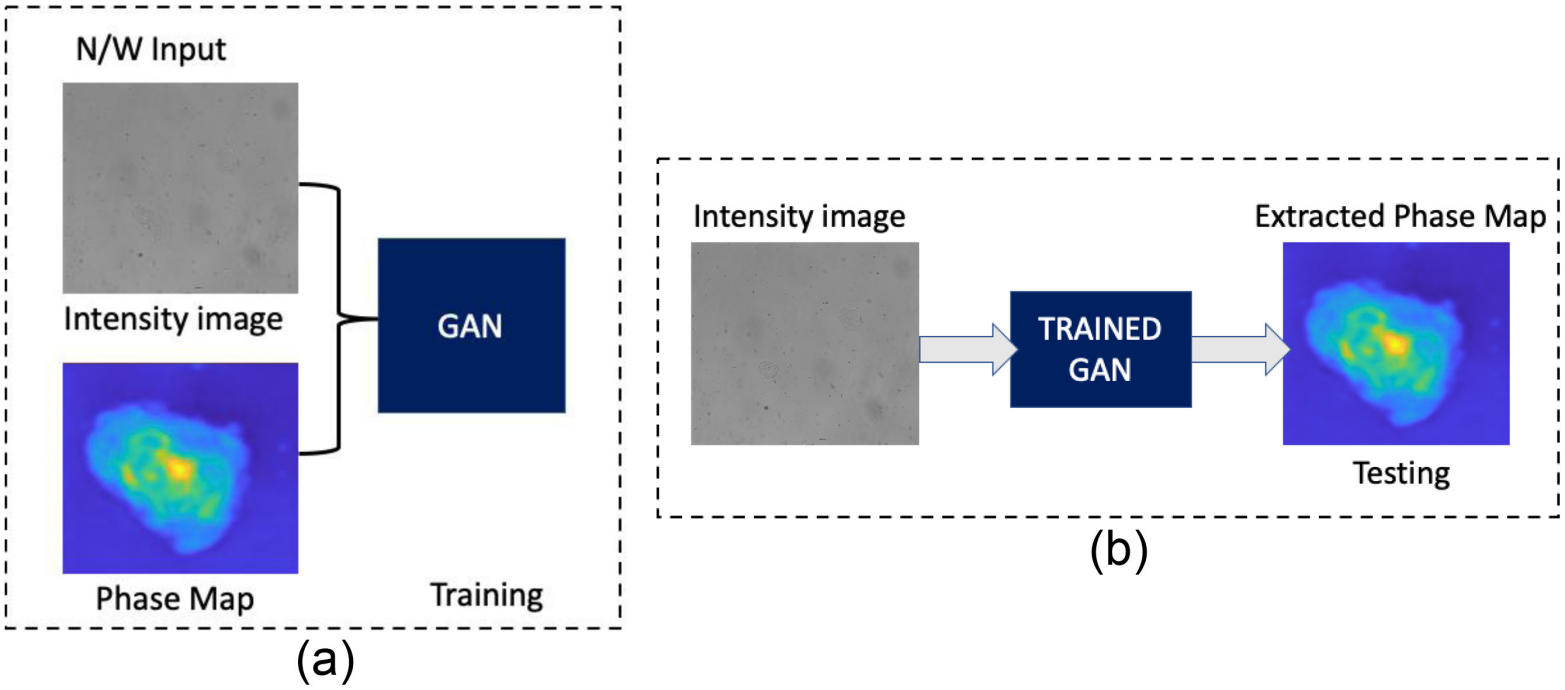
(a) Training methodology of TIE-GANs, (b) the testing methodology and network output as phase image.

Following dataset development, GANs are used to train image pairs consisting of an intensity and phase map. The network’s output is a phase map that corresponds to the input image’s intensity after proper training. Our approach incorporates data from a variety of focal lengths and positions along the optical axis in z direction to strengthen the resulting model. The training and testing procedure for the neural network is shown in Figs. 4(a) and 4(b).

**Fig. 5:**
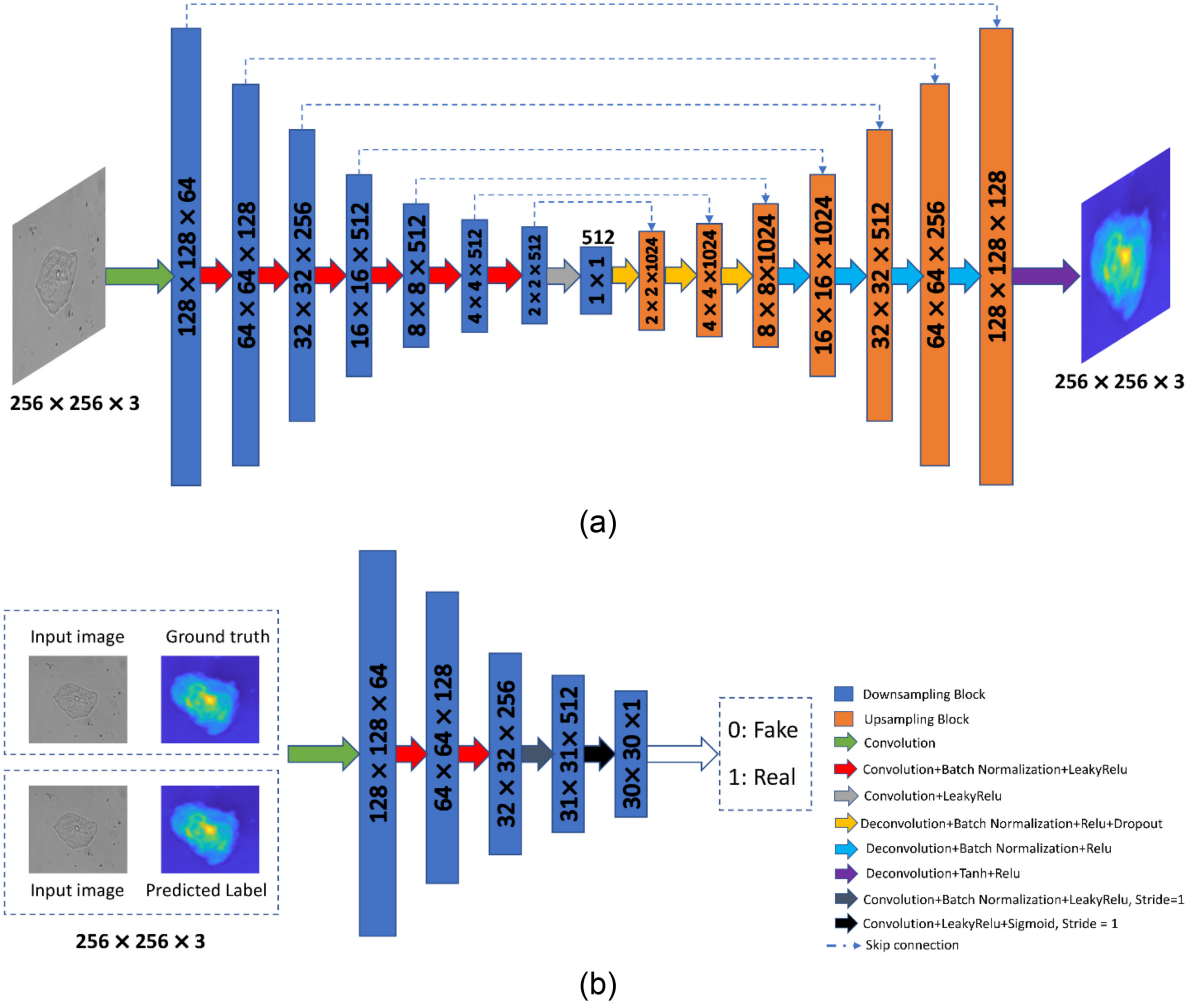
(a) Generator network to generate images from random noise vectors. (b) Discriminator network.

In our approach, the TIE-GANs are based on cGANs architecture consisting of two networks: generator and discriminator. The generator is inspired from U-Net architecture, which is widely accepted in biomedical image segmentation.^67^ The network proposed here uses the encoder–decoder scheme at the generator side with skip connections.^64^ The input image is downsampled using eight convolutional layers in the encoder part followed by eight upsampling layers in the decoder part.^68^ The activation function used after every encoder node is Leaky ReLu.^69^ In order to avoid the overfitting during training of the neural network, we have implemented the dropout at the decoder part. Batch normalization has been used after each layer in order to make the input to each network standardized. The schematic of the network architecture used for generator is shown in Fig. 5(a). Discriminator network follows the PatchGAN structure where the input to the network is a pair of real set and generated set of images. The primary goal of this network is to determine whether or not the produced image is genuine. The discriminator takes on a new function by instructing the generating network on how similar the data should be to real-world images. The picture pair is encoded into a feature vector by the discriminator’s five convolutional layers. After sigmoid activation functions are applied to the last layer of the network, a binary output of 0 or 1 is produced, representing an actual picture pair. The schematic of the discriminator network is shown in Fig. 5(b).

### 3.4 Accuracy Assessment of TIE versus TIE-GANs

The SSIM, MSE, PSNR, and the UIQI are used to measure the quality of an image with respect to ground truth images.

The MSE is the first parameter to be considered because it is widely used and its calculation is easy and inexpensive. One of the primary characteristics of the MSE is its independence, which makes it memoryless and therefore capable of being evaluated on each sample. The MSE’s physical significance is that it is a measure of signal fidelity in terms of the error signal’s energy.

The MSE was calculated between translated, i.e., GANs output image and ground truth TIE image, if $x=\{x_{i}|1,2,\ldots ,N\}$ and $y=\{y_{i}|1,2,\ldots ,N\}$ two signals i.e., images the MSE can be calculated as^70^

$$\mathrm{MSE}(x,y)=\frac{1}{N}\overset{N}{\underset{i=1}{\sum }}{\left(x_{i}-y_{i}\right)}^{2},$$ (8)

where N represents the number of image samples.

The PSNR is one of the most used objective methods for quality assessment metric for translated and ground truth image.^71^ High PSNR value implies a high-quality translated image as the MSE between translated and ground truth image becomes minimum.

The further extension to the MSE leads to the PSNR calculation, which can be represented as

$$\mathrm{PSNR}=10{\;\log}_{10}\;\frac{L^{2}}{\mathrm{MSE}},$$ (9)

where $L^{2}$ is the dynamic range of the image.

The UIQI is applied since the performance of MSE and PSNR does not incorporate luminance as well as contrast factor of the images. UIQI is a unique parameter that gives the image distortion in terms of three factors: loss of correlation, luminance distortion, and contrast distortion.^72^

The UIQI is the measure of three parameters so it can be expressed as

$$\mathrm{UIQI}=\frac{\sigma_{xy}}{\sigma_{x}\sigma_{y}}\cdot \frac{2\mu_{x}\mu_{y}}{{\left(\mu_{x}\right)}^{2}+{\left(\mu_{y}\right)}^{2}}\cdot \frac{2\sigma_{x}\sigma_{y}}{\sigma_{x}^{2}+\sigma_{y}^{2}}.$$ (10)

The first term represents the correlation coefficient between image X and Y, the dynamic range of this factor is [−1,1].

The second term represents how close the luminance is in between both the images, the value of this factor ranges between [0,1]. The third term represents the contrast and gives the similarity factor between both input and translated images, the value of this factor ranges between [0,1]. Hence by combining all these parameters, the UIQI can be expressed as

$$\mathrm{UIQI}=\frac{4\sigma_{xy}\mu_{x}\mu_{y}}{\left(\sigma_{x}^{2}+\sigma_{y}^{2})[{\left(\mu_{x}\right)}^{2}+{\left(\mu_{y}\right)}^{2}\right]}.$$ (11)

Above all the quality assessment metrics performs well but do not consider the human visual system (HVS). The HVS perception is based on the structural information of the scene, and evaluation based on structural loss is vital in terms of quality assessment. The SSIM index is a well-known parameter to compare the structural similarity between two reconstructed and ground truth images. It gives the quality measurement in terms of luminance, contrast, and structure, SSIM is a widely used and accepted parameter for quality assessment.^73^^,^^74^

The SSIM between two images X,Y can be calculated as

$$\mathrm{SSIM}(X,Y)=\frac{\left(2\mu_{x}\mu_{y}+C_{1})(2\sigma_{xy}+C_{2}\right)}{\left(\mu_{x}^{2}+\mu_{y}^{2}+C_{1})(\sigma_{x}^{2}+\sigma_{y}^{2}+C_{2}\right)}.$$ (12)

For UIQI and SSIM, $\mu_{x}$ and $\mu_{y}$ the average image intensity of images X and Y, respectively, $\sigma_{x}^{2}$ and $\sigma_{y}^{2}$ are the variance of both the images. $\sigma_{xy}$ is the cross-covariance of images, $c_{1}=(K_{1}L{)}^{2}$, $c_{2}=(K_{2}L{)}^{2}$ are the stabilization constant, where $K_{1}$ and $K_{2}$ is smaller than 1 and L is the dynamic range of the image.

## 4 Results and Discussions

**Fig. 6:**
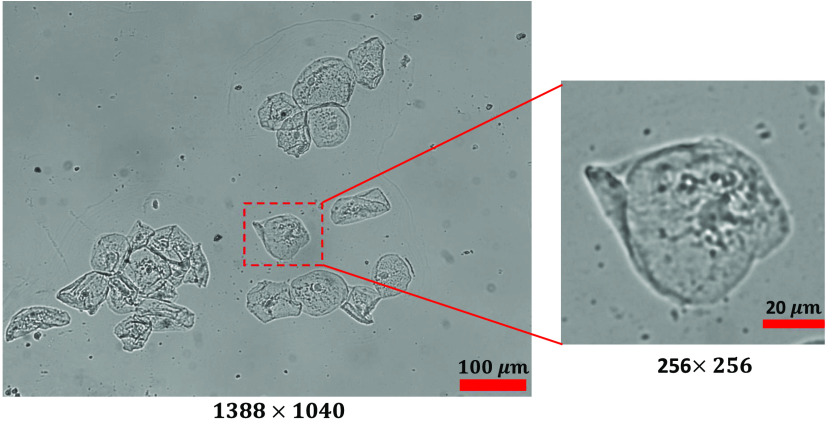
Captured intensity image and extracted ROI for training.

**Fig. 7:**
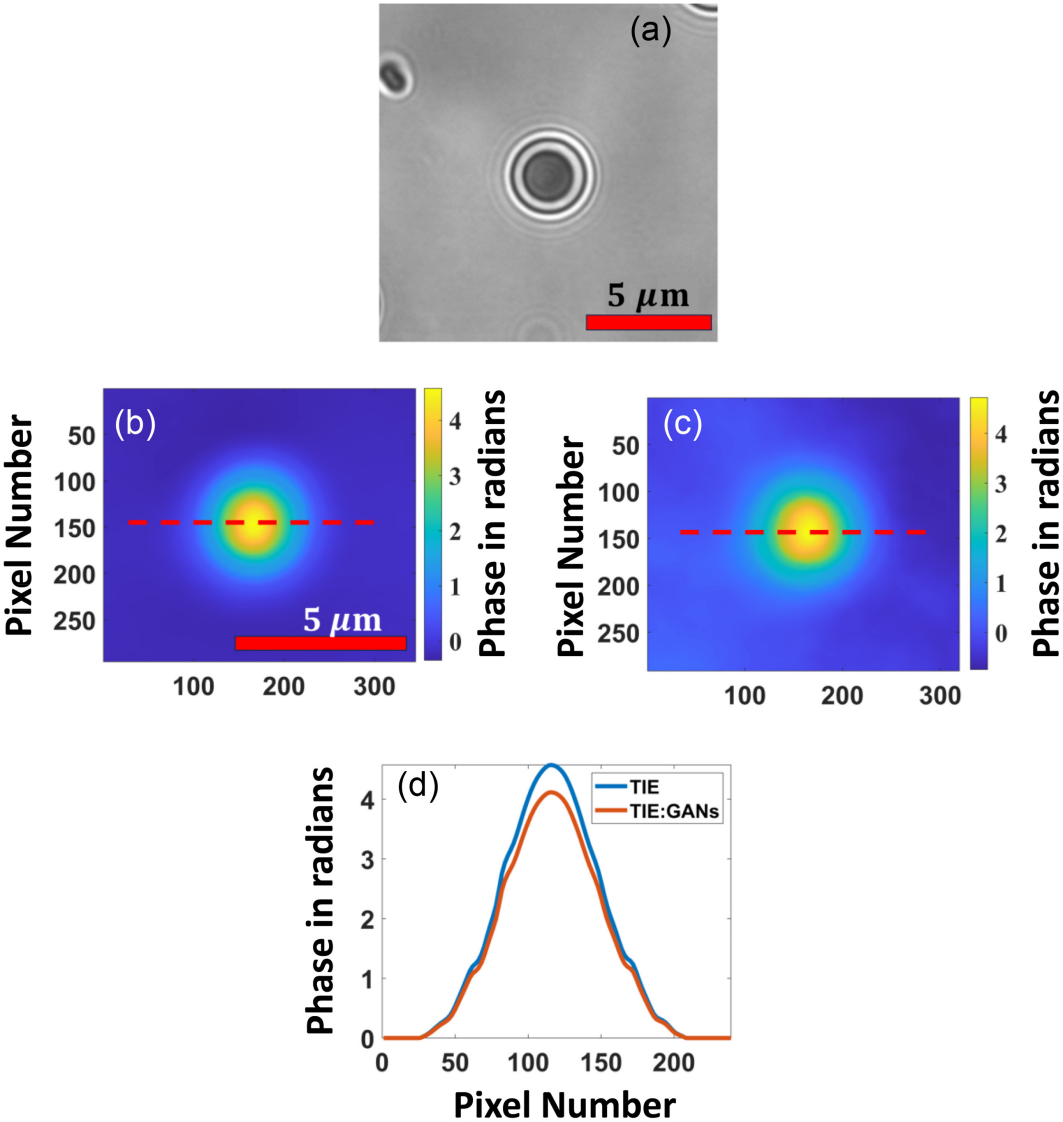
(a) Input intensity image, (b) TIE phase reconstruction, (c) proposed TIE-GANs reconstruction, (d) phase profile for TIE and proposed TIE-GANs methods.

One of the findings during our testing is that if we increase the ROI bounding box having multiple cells for training in the region, it increases the time of training, but the SSIM and other parameter do not show any significant improvements. The captured image and extracted ROI images are shown in Fig. 6. Hence, the experiment was conducted on images having single oral cell, with a size of 256×256  pixels. The dataset contains 700 images for training and 100 for testing and 100 images for validation. Similarly for the characterization, the microbeads are used with known refractive index and thickness. The proposed TIE-GANs were used to reconstruct the phase image of microbeads measuring 4  μm in size (Sigma-Aldrich) with a known refractive index of 1.68 for characterization. The specimens were immersed in distilled water with a refractive index of 1.33, which has a linear attenuation coefficient of $0.843\;{\mu m}^{-1}$. The results in Fig. 7(a) show input intensity image, Fig. 7(b) depicts the phase reconstruction using TIE, Fig. 7(c) represents the proposed TIE-GANs reconstruction, and Fig. 7(d) shows phase profile for both methods. The characterization data showed 0.98 SSIM value, which clearly verifies the validity of our proposed method with microbeads.

**Fig. 8:**
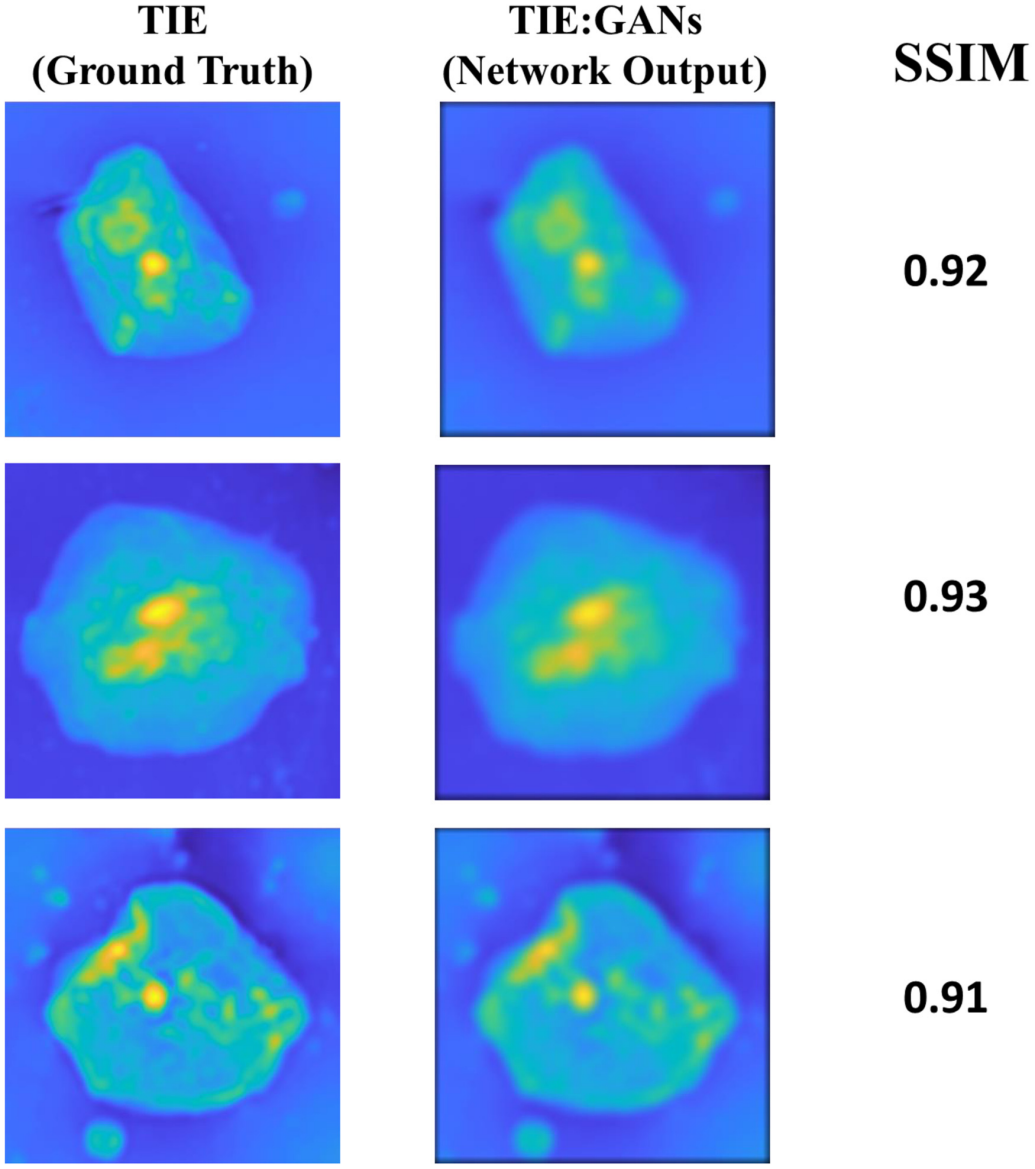
Conventional TIE and TIE:GANs reconstruction with SSIM values.

The initial learning rate and batch size were set to be at 0.0001 and 4, respectively, and the optimizer used was Adam, which is based on the stochastic gradient descent.^75^ Our method performed two sets of iterations, one with 600 images and 200 epochs and the second with 700 images and 300 epochs. After 300 epochs, the network seems to be saturated and does not show any significant increase in accuracy to produce high SSIM reports. Figure 10(a) shows the results with 600 images and 200 epochs where we observed the higher number of SSIM were reported to that of the ground truth images. While in Fig. 10(b), the SSIM shows the data lying toward the 0.75 range, which also depicts after certain epochs despite the increase in the number of images the network becomes saturated, i.e., similarity to the ground truth data becomes less. The results from both the experiments are shown in Fig. 8. The maximum SSIM observed during our experiment was found to be 0.95 with 600 images and 200 epochs.

The detailed results of SSIM index are presented in Table 1 for both the experiments. We observed that after 200 epochs the network started losing its capability to generate new samples near to actual ground truth images gradually. The experimental parameters of both the experiments have been shown in Table 1.

**Table 1:** Experimental parameters.

Parameter	Experiment 1	Experiment 2
Number of images	600	700
Epochs	200	350
Max SSIM	0.95	0.94
Min SSIM	0.78	0.76
Max MSE	1364.98	1856.61
Min MSE	150.14	148.20
Max PSNR	26.36 dB	26.42 dB
Min PSNR	16.78 dB	15.45 dB
Max UIQI	0.89	0.90
Min UIQI	0.33	0.42
Time taken	1 h	1.5 h

**Fig. 9:**
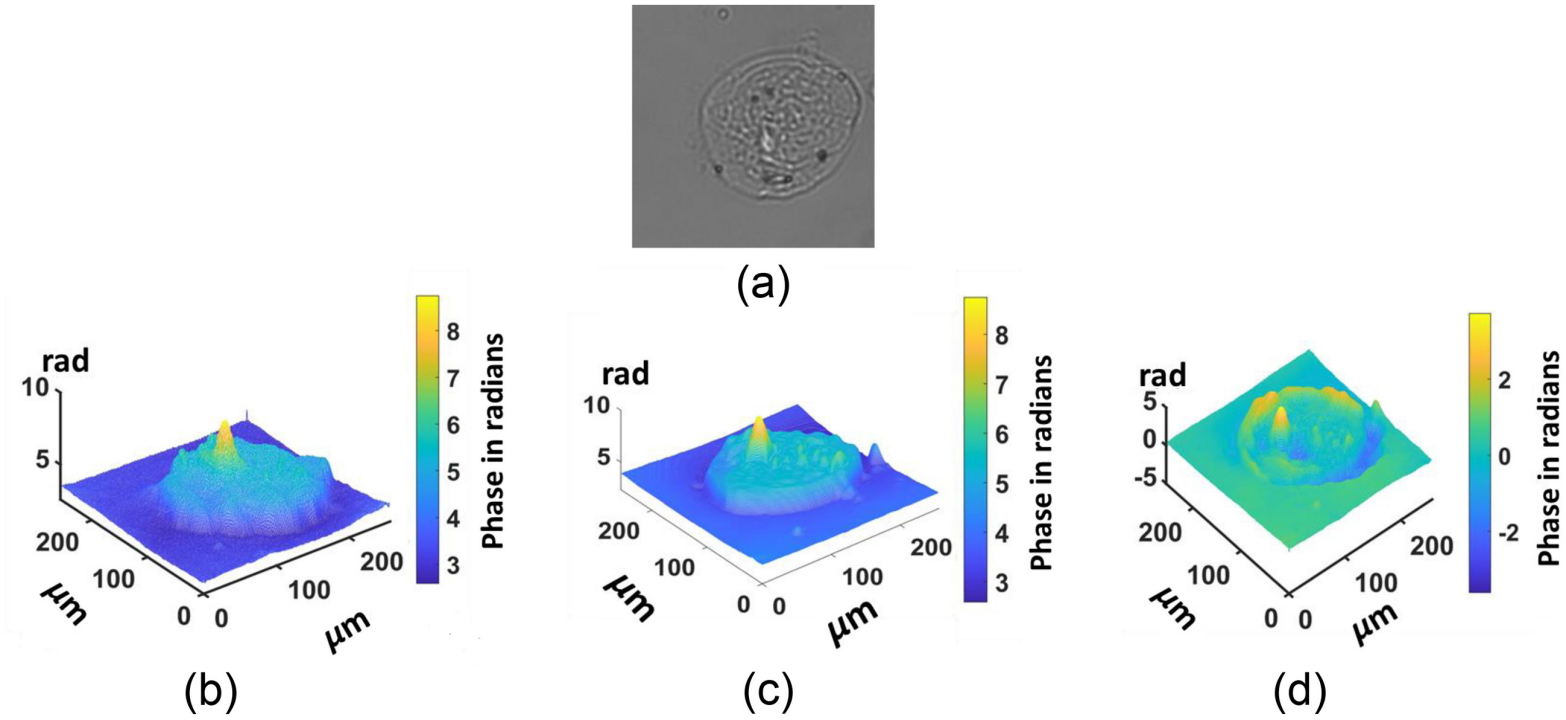
Reconstruction results of TIE-GANs. (a) Input defocused intensity image, (b) ground Truth data conventional TIE, (c) TIE-GANs output network generated output, and (d) error map between ground truth and TIE-GANs.

We calculated the various parameters based on these observations and found that the experiment works best with 200 epochs and around 600 images. Figure 9(a) shows the intensity image, which is the input to the TIE: GAN’s network, Fig. 9(b) shows the ground truth image. We can observe TIE-GANs output in Fig. 9(c), which clearly depicts the quantitative phase map and Fig. 9(d) shows the error map between TIE-GANs and ground truth image.

One more factor that is visible during training process is that along with the high number of images we need to introduce robustness to the network as well to avoid overfitting.^76^ To do that, a skip connection was introduced in the network. However, it is observed that it is highly uncertain to determine the optimized parameter to get the desired results. The parameter needs to be tested through several iterations until the likely output is achieved. Overfitting of the network is one of the problems that arise during training. Our method utilizes skip connection and dropout in the deconvolution part of the network to avoid overfitting. TIE-GANs are found to be effective, and their execution time after training is as low as ∼0.010  s for generating phase map image. We do expect that the training cycles, the volume of training data (number of phase images) that is needed for practically reconstructing objects with different phases and thicknesses will be much high compared to what is demonstrated as a proof of concept in this paper. We have used beads of thicknesses ∼4  μm with a microscope objective magnification of (63×, 1.4 NA) in one set of images. We found that a defocus distance of 1  μm was optimal for this NA and magnification. A microscope objective (40×, NA: 0.65) was used for imaging the exfoliate buccal cells. Light source exposure of the camera settings has been adjusted according to the sample for good quality images.

To introduce randomness to the network during training process, some of the images given to the network is unpaired, so the network does not get saturated over time. Although, unpaired image dataset is a well-known method that is known as cycle GANs.^77^ During our training process, we observed that introducing false image pair increases the accurate image pair generated.

Our method is quite simple, and it works on the defocused images and the defocus distance that is trained during training of the samples. During testing, a single intensity defocused image is input to the algorithm and without any decision on the best focus plane or numerical iterative wave propagations, we retrieve the best phase image. This saves time and computational labor. The one to one mapping of pixels from the input defocused object to the output phase object can be elegantly achieved through training using GANs for a larger defocused distance, which is an advantage of TIE:GANs. Another important advantage of our technique is that the proposed algorithm works on DL derived GANs networks. Hence unlike other iterative phase retrieval algorithms, we retrieve the phase image instantly from the trained network. This gives us a remarkable edge in terms of real time operations compared to most other iterative and numerical propagation techniques.

**Fig. 10:**
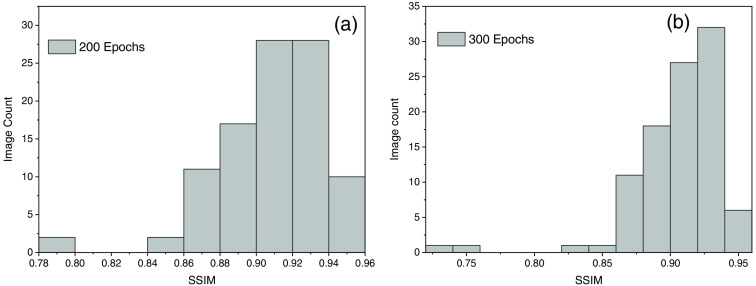
(a) Figure with 200 epoch and 600 image sample size. (b) SSIM histogram with 300 epoch and 700 image sample size.

Figure 10(a) shows the SSIM histogram for both experiments, which reveals that the first iteration with 200 epochs has a greater percentage of SSIM values in the range of 0.92 to 0.94. Figure 10(b) for iteration 2 with 300 epochs also displays values near the 0.90 range, but it is evident that it has a low SSIM index compared to iteration 1. Maximum SSIM reported with 200 epochs and 600 image sample size was 0.95. Therefore, TIE-GANs-based reconstruction can reconstruct images with 95% similarity to the conventional TIE scheme, i.e., ground truth images.

**Fig. 11:**
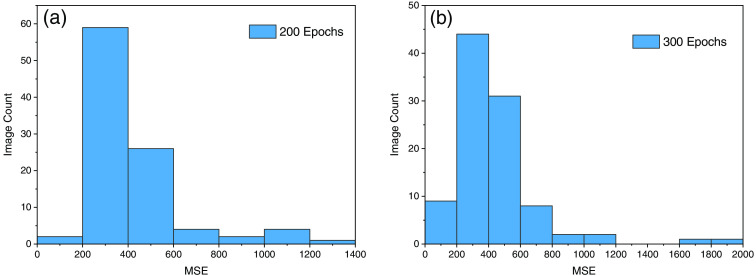
(a) MSE value for 200 epochs and 600 sample. (b) MSE for 300 epochs and 700 image sample.

The second parameter for quality assessment for reconstruction is MSE, which is calculated using Eq. (8) the histogram and the MSE distribution is shown in Figs. 11(a) and 11(b).

MSE computes the average squared error between each pixel in the two images being compared. From Fig. 11(a) with 200 epochs, we can observe that the maximum MSE is coming around the 1400 range. While in the second experiment it is exceeding the 2000 range means that the value of MSE is quite high as compared to the first iteration. Since we are looking for lower number of MSE hence first experiment makes the optimum sense regarding this experiment. Because the maximum number of values lies between 200 and 400 range, the result shows that the error is low as expected and it shows closeness to the ground truth images.

The PSNR value calculated using Eq. (9) and the maximum value for 200 epochs was found to be 26.36 dB and for 300 epochs with 700 training image pair the maximum value found to be 26.42 dB. The experimental parameter for PSNR is shown in Table 1.

**Fig. 12:**
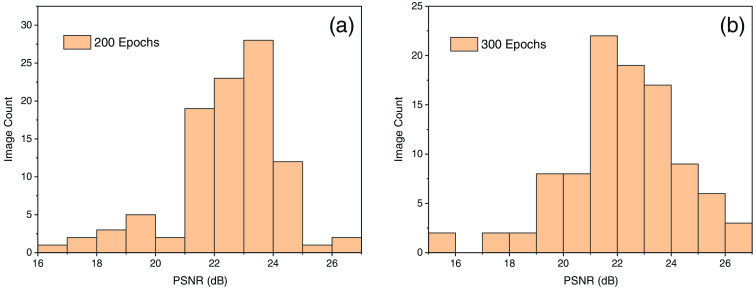
(a) PSNR value for 200 epochs and 600 image sample. (b) PSNR for 300 epochs and 700 image sample.

There was no such significant increase observed from 200 to 300 epochs hence it verified our observation with SSIM that after 200 epochs network start saturating. The histogram and the distribution of the PSNR value on test images are shown in Figs. 12(a) and 12(b).

**Fig. 13:**
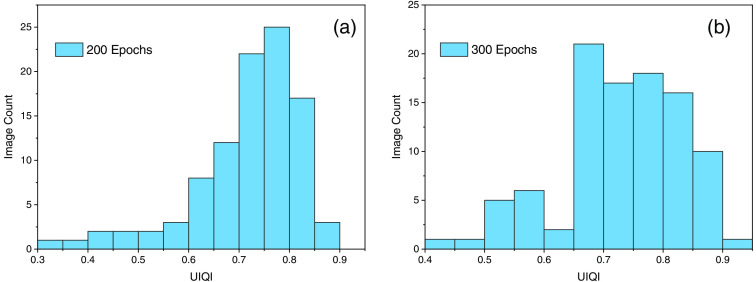
(a) UIQI value for 200 epochs and 600 image sample. (b) UIQI for 300 epochs and 700 image sample.

The final parameter for consideration is UIQI, which is calculated using Eq. (11). The UIQI is the combined measure of correlation, luminance, and contrast, and the histogram for epochs 200 and 300 and distribution of values is shown in Figs. 13(a) and 13(b). The UIQI ranges between 0 and 1, 0 shows no similarity between two images and 1 shows identical images to the ground truth images. The maximum UIQI observed is 0.90 for experiment 2, although the experiment 1 also gave significant results and reached about 0.89 UIQI value. From these four parameters, we came to the conclusion that despite of increment in the number of images in the second experiment the first one with 600 images worked well for optimum results.

**Fig. 14:**
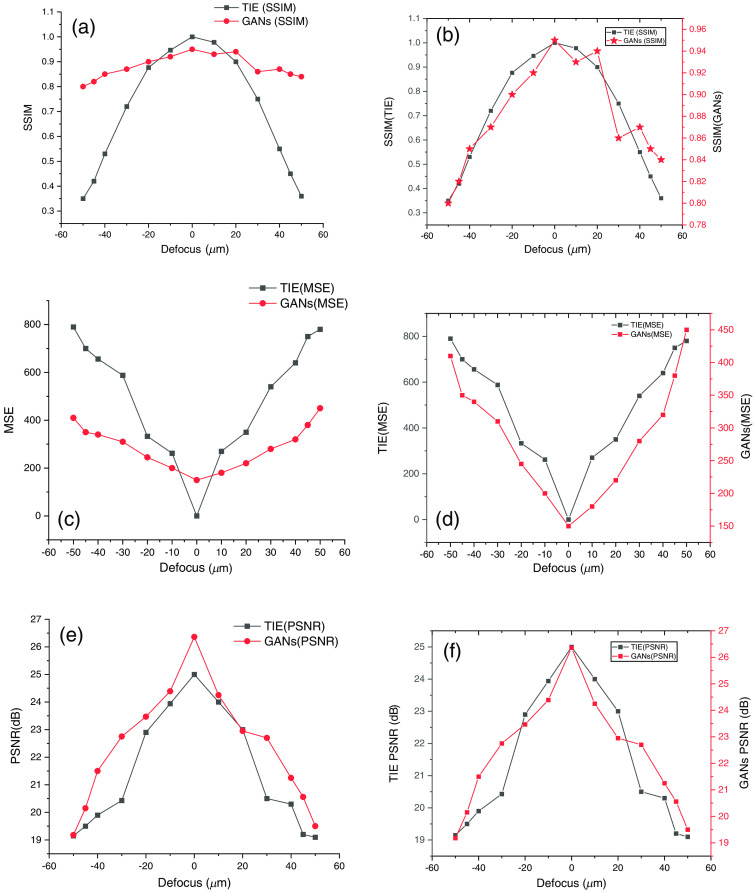
(a), (b) SSIM with conventional TIE versus TIE-GANs; (c), (d) MSE TIE versus TIE-GANs; (e), (f) PSNR TIE versus TIE-GANs.

Since the conventional TIE is not a single shot scheme, it requires multiple images to be recorded at different defocus planes provided that the sample is non-homogeneous. Since we cannot have a single shot scheme for non-homogeneous samples, one of the advantages of our method is that it can work on any sample despite its nature. For non-homogeneous samples, the conventional TIE works in such a way we must find the best infocus image from intensity image stack using tamura coefficient as mentioned in the methodology section. Afterward it demands to compute SNR value by putting two defocus images one from overfocus and one from underfocus plane for phase reconstruction. This process always takes tedious parameter calibration. However, in the TIE-GANs dataset has been prepared using the different defocus plane images with oral cells so that it learns the features from different defocus distances during training itself. Figures 14(a) and 14(b) show the SSIM reconstructed at different defocus planes, which shows the SSIM reconstructed using conventional TIE and TIE-GANs. It is observed that if we propagate through the different defocus planes the TIE-GANs are able to produce phase maps closer to ground truth. As we shift away from the infocus plane and reconstruct phase map using three defocused images, the SSIM decreases gradually in case of conventional TIE shown in Fig. 14(a). In the case of conventional TIE, it ranges from [0.35, 1] while in case of TIE-GANs it never reaches the value of 1 but it never drops below 0.80 range. It shows the consistency of the TIE-GANs to reconstruct phase map over long defocus distances as shown in Figs. 14(a) and 14(b).

Similar results can be observed for the MSE and PSNR values as well. In Figs. 14(c) and 14(d), it is clearly observed that the conventional TIE MSE can be ranged between [0,800], whereas TIE-GANs range between [150,450]. So, we can conclude that the TIE-GANs results have consistency over reconstruction in terms of accuracy. It produces minimal noise as low as possible even if we propagate along the optical axis in the Z direction. The final parameter for consideration is the PSNR value we can observe that in Figs. 14(e) and 14(f) the maximum PSNR value in terms of the TIE-GANs reaches up to 26.42 dB while in case of conventional TIE it reaches the value of the 24.5 dB, the values here even exceed the conventional TIE value. The minimum value that we observed during our experiment was 19 dB in the conventional TIE while in the case of the TIE-GANs it never drops below 19.5 dB. From comparative analysis of all these parameters, we can conclude that although conventional TIE performs well for non-homogeneous samples, TIE-GANs can perform well as a single-shot scheme. The TIE-GANs can reconstruct phase for long defocus distance in optical axis. Hence, TIE-GANs and other related schemes for the phase reconstruction based on DL hold the future for phase contrast microscopy. The challenge that one has to face during implementation is that we need to train the network with the same sample then only we are able to get the desirable results. In the case of conventional TIE, one does not have to hold the knowledge of the sample beforehand the mathematical implementation of the algorithm does the job for phase reconstruction. In case of TIE-GANs, the network must learn the feature map of the sample through training. After training, only TIE-GANs can predict feature map for the phase image and works as a single shot technique. Although unpaired image translation techniques have potential for phase reconstruction, we have not seen better statistical improvements as of now hence did not consider them for reconstruction in this study.

## 5 Conclusion

The incorporation of QPI with DL methodologies is a noteworthy achievement within the domain of microscopy and data processing. The convergence of these two innovative disciplines has the potential to fundamentally transform our understanding of biological systems, materials, and beyond. The label-free feature of QPI and DL algorithms enables automatic extraction of complicated information, enabling more accurate and efficient analysis. This study has emphasized the novelty and benefits of using DL algorithms, such as TIE-GANs for quantitative phase microscopy. The qualities of TIE such as label-free nature and less complex experimental set-up works as an add-on to QPI analysis with DL. This study is able to employ a method based on GANs to reduce the necessity of manual labor. Our method solved complexity in capturing a large number of images at different defocus planes by enabling single shot technique. The utilization of GANs as a methodology has been found to be proficient in generating phase images, provided that the dataset has been suitably prepared. TIE-GANs are able to convert single-intensity images to phase maps in rapid manner, and the results are encouraging for oral cells. The characterization is done with microbeads, which produces 98% SSIM and improved PSNR value. When trained on a structured dataset, as demonstrated by the microbead validation results, the proposed method can be used to characterize nanometric samples as well. The SSIM for oral cell was reported to be 95% and there is room for further improvement when the network is fine-tuned. There are some demerits of DL in terms of computation as it requires a heavy hardware setup. High configuration requirements make these algorithms and experiments data-, and computation-hungry. However, the future of artificial intelligence holds great potential along with QPI and other sectors of biomedical imaging. One of the key findings during our experiment was that it works for images on any defocus distance up to ±60  μm in z direction along the optical axis. The novelty of our approach showed that even if we provide intensity image at any distance in optical axis, and it is able to reconstruct the phase map of these intensity images under provided range. Future work for this research includes the accuracy assessment as well as tuning of hyperparameters that will improve our current parameters. Another dimension of this research is to extend this similar capability for lenless holographic system as well for the virtual staining of the samples based on thickness or other properties. The future work for this research consists of the unpaired image translation for phase reconstruction. The future work of our method includes the nanometric resolution characterization for RBC and sperm cells.

## Data Availability

All relevant data, materials, and software code used in this research are available upon request from the corresponding author.
